# Analysis of propionate‐degrading consortia from agricultural biogas plants

**DOI:** 10.1002/mbo3.386

**Published:** 2016-07-01

**Authors:** Stephan Ahlert, Rita Zimmermann, Johannes Ebling, Helmut König

**Affiliations:** ^1^ Institute of Microbiology and Wine ResearchJohannes Gutenberg UniversityMainzGermany

**Keywords:** biogas, propionate, degradation, syntrophy, community, methanogens, homoacetogens

## Abstract

In order to investigate the propionate‐degrading community of agricultural biogas plants, four propionate‐degrading consortia (Ap1a, N12, G12, and Wp2a) were established from different biogas plants which were fed with renewable resources. The consortia were cultivated in a batch for a period of 2–4 years and then analyzed in an 8‐week batch experiment for microbial succession during propionate degradation. Community shifts showed considerable propagation of *Syntrophobacter sulfatireducens*,* Cryptanaerobacter* sp./*Pelotomaculum* sp., and “*Candidatus* Cloacamonas sp.” in the course of decreasing propionate concentration. Methanogenic species belonged mainly to the genera *Methanosarcina*,* Methanosaeta,* and *Methanoculleus*. Due to the prevalent presence of the syntrophic acetate‐oxidizing species *Tepidanaerobacter acetatoxydans* and potentially autotrophic homoacetogenic bacteria (*Moorella* sp., *Thermacetogenium* sp.), a theoretical involvement of syntrophic acetate oxidation and autotrophic homoacetogenesis in stable and efficient propionate degradation was indicated. Considering theoretical Gibbs free energy values at different hydrogen partial pressures, it is noticeable that syntrophic acetate oxidation and autotrophic homoacetogenesis have the potential to counterbalance adverse hydrogen partial pressure fluctuations, stabilizing most probably continuous and stable propionate degradation.

## 1 Introduction

Global energy consumption is constantly rising due to the ongoing industrial development of the world's largest economies and the uprising emerging markets (International Energy Agency, 2013). The growing demands for energy resources at the present time are mainly met by fossil energy resources. These are derived from oil, coal, and natural gas, which release formerly bound carbon as carbon dioxide into the atmosphere (British Petroleum, 2014). Since the accumulation of this greenhouse gas into the atmosphere contributes highly to global warming and climate change (Solomon, Plattner, Knutti, & Friedlingstein, 2009), leading economies have decided to reduce the release of fossil‐bound carbon dioxide through the use of renewable resources, such as solar energy, wind power, and energy from biomass degradation. Biomass consists of vegetational‐bound solar energy, which does not increase atmospheric carbon dioxide concentration, because of the carbon cycling nature of plants. Thus, energy production from biomass degradation is considered to be neutral with respect to climate change (Srirangan, Akawi, Moo‐Young, & Chou, 2012).

According to the European Biogas Association, new biogas plants have been installed all over Europe totaling nearly 15,000 plants in 2013 (European Biogas Association, 2014). The increase in biogas production implicates the need for technological optimization of the microbial degradation of plant material. Therefore, many research projects have widened the knowledge available concerning the microbiology of biogas‐producing biomass degradation (Krakat, Westphal, Schmidt, & Scherer, 2010; Theuerl et al., 2015; Wirth et al., 2012). In this respect, one of the challenges is the control of the propionate concentration by investigating microbial propionate degradation. Up to now, studies concentrating on propionate degradation in agricultural biogas plants have been underrepresented.

Propionate is a common fermentation product during the degradation and fermentation of biomass to biogas. There is a constant turnover in stably operating biogas plants (Noll, Klose, & Conrad, 2010). However, operational mismanagement (e.g., overloading) or inadequate substrate compositions (inhibitor substances, growth factor deficiencies) can hamper the process, leading to propionate accumulation, which aggravates the complications through lowering the pH and microbial inhibition (Fachagentur Nachwachsende Rohstoffe e.V., 2013; Karlsson et al., 2012; Nielsen, Uellendahl, & Ahring, 2007). Therefore, propionate degradation is a limiting factor of anaerobic fermentation (Deublin & Steinhauser, 2008).

The accumulation of propionate is especially challenging, due to the thermodynamic constraints of its degradation. Under standard conditions, propionate degradation is an endergonic process, ΔG^0^′ = +76.1 kJ per reaction (Thauer, Jungermann, & Decker, 1977).

$${\text{Propionate}}^{-}+3H_{2}O\to {\text{Acetate}}^{-}+{\text{HCO}}_{3}^{-}+H^{+}+3H_{2}$$

Considering more appropriate conditions (37°C, 1 mmol L^−1^ acetate and propionate, 20 mmol L^−1^ HCO_3_^−^, 10^−4^ atm H_2_), the degradation exceeds thermodynamic equilibrium and is slightly exergonic, ΔG′ = −5.4 kJ/reaction (Zinder, 1984). This thermodynamic shift results mainly from the lower hydrogen partial pressure, being 1 atm under standard conditions and 10^−4^ atm in the adjusted calculations (Zinder, 1984). Thus, the oxidation of propionate depends on stable hydrogen consumption (or respective electrons) by associated processes (Stams & Plugge, 2009). Under artificial culture conditions, these processes can be triggered through poised‐potential amperometric culture systems (Emde & Schink, 1990), electron scavenging cosubstrates (Stams, Van Dijk, Dijkema, & Plugge, 1993) or flushing (Scholten & Conrad, 2000). However, methanogenic environments depend on hydrogen‐consuming microorganisms, which dispose of hydrogen in syntrophic cooperation (Schink & Stams, 2013). Hydrogenotrophic methanogens especially are often considered as optimal partner organisms for propionate‐degrading bacteria. Culture collections, for instance, offer different isolated propionate‐degrading bacteria in combination with *Methanospirillum hungatei*. However, sulfate‐reducing or homoacetogenic bacteria can also be involved in syntrophic hydrogen utilization (Dong, Plugge, & Stams, 1994; Meng, Zhang, Li, & Quan, 2013; Muyzer & Stams, 2008). The oxidation of propionate to acetate and methane by syntrophic hydrogenotrophic methanogens is an exergonic process, even under standard conditions (ΔG^0^′ = −25.2 kJ per reaction).

$${\text{Propionate}}^{-}+0.75H_{2}O\to {\text{Acetate}}^{-}+0.75{\text{CH}}_{4}+0.25{\text{HCO}}_{3}^{-}+0.25H^{+}$$

In addition, the complete conversion of propionate to methane and carbon dioxide (ΔG^0^′ = −56.6 kJ per reaction) requires the formation of methane from acetate by acetoclastic methanogens (Stams, 1994). Such triple‐cultures may degrade propionate more efficiently than cocultures (Dong et al., 1994).

$${\text{Propionate}}^{-}+1.75H_{2}O\to {\text{Acetate}}^{-}+1.75{\text{CH}}_{4}+1.25{\text{HCO}}_{3}^{-}+0.25H^{+}$$

Isolated and metabolically analyzed propionate‐oxidizing bacteria belong to the phyla of gram‐positive firmicutes (*Desulfotomaculum*,* Pelotomaculum*) and gram‐negative *δ*‐proteobacteria (*Smithella*,* Syntrophobacter*). A total of 10 species from four genera have been described (Li, Ban, Zhang, & Jha, 2012). Syntrophic propionate‐degrading community analyses of rice field soil and municipal or molasses wastewater detected these genera repeatedly (Ariesyady, Ito, Yoshiguchi, & Okabe, 2007; Ban, Zhang, & Li, 2015; Ban et al., 2013; Gan, Qiu, Liu, Rui, & Lu, 2012; Lueders, Pommerenke, & Friedrich, 2004). Based on an ecogenomic analysis, further propionate‐oxidizing species are expected within the candidate divisions Atribacteria and Cloacimonetes (Nobu et al., 2015). The genomic analysis of so far unculturable “*Candidatus* Cloacamonas acidaminovorans” (unclassified bacteria, Cloacimonetes) led to the discovery of all the genes involved in syntrophic propionate degradation (Pelletier et al., 2008). As the growth rates of artificially composed propionate‐degrading cocultures are extremely low (De Bok et al., 2005; Imachi et al., 2002, 2007), further associated species can be assumed for optimal propionate consumption. Accordingly, the addition of *Proteiniphilum* *acetatigenes* to cocultured *Syntrophobacter sulfatireducens* and *Methanobacterium formicicum* accelerated propionate degradation, although the mechanism responsible remained unresolved (Chen & Dong, 2005). A transcriptomic analysis of the syntrophic propionate‐degrading coculture of *Pelotomaculum thermopropionicum* and *Methanothermobacter thermautotrophicus* suggests wider metabolic interrelationships during propionate oxidation, such as amino acids transfer (Kato, Kosaka, & Watanabe, 2009; Sieber, McInerney, & Gunsalus, 2012).

In order to get a deeper insight into the propionate degradation of biogas plants, this study investigated the microbial compositions of propionate‐degrading consortia from agricultural biogas plants fed with renewable resources.

## 2 Materials and Methods

### 2.1 Propionate‐degrading consortia

Fermenter sludge was taken from four German agricultural biogas plants (BGP), which were fed with maize silage/grass silage/cow dung (BGP Arenrath and BGP Oberthal, Germany), maize silage/cow dung (BGP Wallhalben, Germany), and maize silage/whole crop silage/pig manure (BGP Steinweiler, Germany). Samples were incubated in biomass medium containing propionate. The biomass medium was prepared anaerobically in an anaerobic glove box (Coy Laboratory Products, Grass Lake, USA; atmosphere 95% N_2_/5% H_2_). It consisted of 0.56 g L^−1^ KH_2_PO_4_, 1.7 g L^−1^ K_2_HPO_4_, 0.5 g L^−1^ cysteine‐HCl × H_2_O, 0.4 g L^−1^ MgSO_4_ × 7 H_2_O, 0.4 g L^−1^ NaCl, 0.3 g L^−1^ NH_4_Cl, 38 mg L^−1^ CaCl_2_, 2 mg L^−1^ FeSO_4_ × 7 H_2_O, 2 mg L^−1^ resazurine, 1.95 g L^−1^ sodium propionate, 0.3 g L^−1^ Na_2_S × 9 H_2_O, 2.5 ml L^−1^ trace element solution SL‐10 (DSMZ‐Deutsche Sammlung von Mikroorganismen und Zellkulturen GmbH), 2.5 ml L^−1^ vitamin solution (DSMZ‐Deutsche Sammlung von Mikroorganismen und Zellkulturen GmbH), 1 ml L^−1^ selenium and tungsten solution (DSMZ‐Deutsche Sammlung von Mikroorganismen und Zellkulturen GmbH), and 1% 0.2 μm filtrated biomass sludge (from BGP Wallhalben). Finally, the pH value was adjusted to 7.5 using 2 M NaOH and the headspace atmosphere was standardized by nitrogen flushing. Incubation took place at 39°C in the dark without shaking. Stable propionate‐degrading consortia were maintained through constant monitoring of propionate degradation and subsequent reinoculation in fresh media for years. Consortia were named Ap1a (BGP Arenrath), G12 (BGP Oberthal), N12 (BGP Wallhalben), and Wp2a (BGP Steinweiler). Cultivation for community analysis was conducted in biomass medium with 10 % filtrated biomass sludge and 2.5 g L^−1^ sodium propionate. These samples were taken after 14 (t_1_), 39 (t_2_), and 56 (t_3_) days of incubation.

### 2.2 Monitoring propionate concentration

The high‐performance liquid chromatography equipment (Shimadzu, Kyōto, Japan) consisted of a control unit SCL‐6B with two reservoir pumps (LC‐6A), autosampler (SIL‐6B), column oven (STH585, Gynkotek‐Göhler), UV detector (SPD10A), and printer (C‐R8A). The liquid phase consisted of 7.3 mmol L^−1^ KH_2_PO_4_ adjusted to pH 2.3 with concentrated H_3_PO_4_. Separation was achieved with the RP column ProntoSIL Spheribond ODS2 (5.0 μm × 250 mm × 4.6 mm). Separations were conducted at 1 ml min^−1^ flow rate, 30°C, 2 μl injection, and 210 nm UV detection. The propionate concentration was determined via standard solution measurements. Methanol (100 %) was used regularly to reconstitute the column performance.

### 2.3 Nucleic acid extraction and domain‐specific 16S rRNA gene amplification

Consortia samples of 2.5 ml were concentrated to <200 μl via centrifugation (5 min, 17,000 g) and transferred to bead tubes of the GeneMATRIX Stool Purification Kit (EURx Ltd., Gdansk, Poland). The procedure was conducted according to the manufacturer's instructions. Extracted DNA was used for domain‐specific 16S rRNA gene amplification. Primers E5F, 5′‐AGAGTTTGATCMTGGCT‐3′ (Dröge et al., 2005) and E1541r, 5′‐AAGGAGGTGATCCANCCRCA‐3′ (Von Wintzingerode, Selent, Hegemann, & Gobel, 1999) were used for bacterial‐specific amplifications and primers Met86f, 5′‐GCTCAGTAACACGTGG‐3′ (Wright & Pimm, 2003) and Ar1530, 5′‐GGAGGTGATCCAGCCG‐3′ (Stantscheff, 2013) for archaeal‐specific amplifications. Reactions were set up with the peqGold Taq all inclusive reaction kit (VWR International GmbH, Erlangen, Germany) in the following compositions: 36.8 μl nuclease‐free DEPC‐treated water, 1 μl MgCl_2_, 1 μl dNTP mix, primers each 2 μl, 5 μl buffer red, 0.2 μl Taq‐Polymerase, and 2 μl template DNA. Bacterial DNA amplification was achieved using the following program. An initial denaturation period of 5 min at 95°C was followed by 20 touchdown cycles (1 min at 95°C, 1 min at 59.3°C with −0.5°C per cycle, 2 min at 72°C), 10 constant cycles (1 min at 95°C, 1 min at 49.3°C, 2 min at 72°C), and 10 min final elongation at 72°C. Archaeal amplification was performed as follows. Again, an initial denaturation period of 5 min at 95°C was followed by 15 cycles with rising annealing temperatures (30 s at 95°C, 45 s at 55°C with +0.1°C per cycle, 90 s at 72°C), 20 constant cycles (30 s at 95°C, 45 s at 56°C, 90 s at °C), and 5 min final elongation at 72°C.

### 2.4 16S rRNA gene cloning

Domain‐specific 16S rRNA gene amplicons were purified by gel electrophoreses (1.5% agarose) and subsequent gel extraction purification using a GeneJet Gel Extraction Kit (Thermo Fisher Scientific, Waltham, USA). The purified fragments were cloned into the pCR4‐TOPO vector using a TOPO TA Cloning Kit For Sequencing and One Shot TOP10 Chemically Competent *E. coli* cells (Life Technologies, Carlsbad, USA). Inserts of 60 bacterial 16S rDNA clones per consortium sample (four consortia, three samples each after 14, 39, and 56 days) and inserts of 12 archaeal 16S rDNA clones of four samples (each consortium after 39 days) were amplified in colony PCR (35.8 μl nuclease‐free DEPC‐treated water, 1 μl MgCl_2_, 1 μl dNTP mix, primers T3/T7 à 1 μl, 5 μl buffer red, and 0.2 μl Taq polymerase; reactants from VWR International GmbH, Erlangen, Germany) according to the following program: 10 min initial denaturation at 95°C was followed by 40 constant cycles (1 min at 94°C, 1 min at °C, 2 min 72°C) and 10 min final elongation at 72°C.

### 2.5 ARDRA analysis, DNA sequencing, and removal of chimeric sequences

The 16S rRNA clones were phylogenetically grouped via ARDRA analysis. All restriction enzymes (10 U μl^−1^) and respective buffers utilized in the ARDRA analysis were obtained from Thermo Fisher Scientific, Waltham, USA. Bacterial 16S rRNA gene clone amplicons derived from colony PCR were cut in two separate reactions with *Hha*I and *Hinf*I restriction enzymes. Bacterial PCR amplicons (8.7 μl) were mixed with 0.3 μl restriction enzyme in 1 μl green buffer and incubated for 5 hr at 37°C. Archaeal ARDRA analysis was conducted according to (Stantscheff, 2013). The 16S rRNA gene clone amplicons derived from colony PCR were cut in two separate reactions, using restriction enzyme *Hae*III and a mixture of the two enzymes *Sma*I and *Xho*I. An amount of 10 μl archaeal PCR amplicons were mixed with 1 μl *Hae*III in 1 μl buffer R and incubated for 1 hr at 37°C. For *Sma*I and *Xho* digestion, 10 μl archaeal PCR amplicons were mixed with 1 μl *Sma*I in 1 μl tango buffer and incubated for 1 hr at 30°C. Then, 1 μl *Xho*I in 1.5 μl buffer R was added for further incubation for 1 hr at 37°C. Restriction patterns of bacterial and archaeal ARDRA analysis were evaluated via gel electrophoresis (2 % agarose) and ethidium bromide DNA staining. One 16S rRNA gene clone out of every ARDRA group (fragments with the same ARDRA pattern) was sequenced by LGC Genomics GmbH, Berlin, Germany. Afterward, DECIPHER's Find Chimeras online tool (http://decipher.cee.wisc.edu/FindChimeras.html) was applied in order to identify and remove the chimeric bacterial 16S rRNA clone sequences.

### 2.6 Community reconstruction

Sequencing (primer T3) of bacterial 16S rRNA clones lead to gene fragments between 747 and 1141 bp covering either the 5′‐ or the 3′‐end of the gene. Fragments covering the 5′‐end were clustered using BLASTclust (80 % query coverage, 97% sequence identity, http://toolkit.tuebingen.mpg.de/blastclust). In order to build a comparable dataset, one 16S rRNA clone out of every cluster was resequenced from the other direction, leading to sequences only covering the 3′‐end of the gene. Subsequently, all 16S rRNA clones covering the 3′‐end of the gene were also clustered as described above. Phylogenetic relationships were determined via NCBI blastn database enquiry (16S rRNA sequences database of bacteria and archaea, https://blast.ncbi.nlm.nih.gov/). Finally, the ARDRA groups and sequencing results were assigned to the source samples and led to bacterial community reconstructions. Archaeal 16S rRNA gene clones were subjected directly to the NCBI blastn database enquiry.

### 2.7 Domain‐specific quantitative real‐time PCR (qPCR)

Quantification of total bacteria and total archaea was determined according to (May et al., 2015), using an artificial DNA fragment for standard preparation and the primer combinations BAC338F/BAC805R and 931F/M1100R for bacterial and archaeal 16S rRNA gene‐fragment amplification. The qPCR assays were performed using a realplex2 ep gradient S Mastercycler (Eppendorf AG, Hamburg, Germany) supported by the evaluation software realplex 2.2. Reactions were carried out using the iQ^™^SYBR^®^ Green Supermix (BioRad, Hercules, USA) applied into white EasyStrip snap tubes (Thermo Fisher Scientific).

### 2.8 Microorganisms and accession numbers

Representative DNA sequences of our bacterial 16S rRNA sequence clusters can be obtained through the following GenBank accession numbers: *Aminobacterium colombiense* Baena, Fardeau, Labat, Ollivier, Thomas et al., (1998b; Chertkov et al., 2010) KT878632, *Caloramator* sp./*Moorella* sp. KT878633/KT878631, “*Candidatus* Cloacamonas sp.” KT878625, *Cryptanaerobacter* sp./*Pelotomaculum* sp. KT878641/KT878634, *Defluviitoga tunisiensis* (Ben Hania et al., 2012) KT878629, *Desulfovibrio aminophilus* Baena, Fardeau, Labat, Ollivier, Garcia et al., (1998a) KT878630, *Mesotoga infera* (Ben Hania et al., 2013) KT878628, *Sedimentibacter* sp. KT878636, *Syntrophaceticus* sp./*Thermacetogenium* sp. KT878635/KT878637, *Syntrophobacter sulfatireducens* (Chen, Liu, & Dong, 2005) KT878627, *Ornatilinea* sp. KT878639, *Tepidanaerobacter acetatoxydans* (Westerholm, Roos, & Schnurer, 2011) KT878626, *Treponema* sp. KT878638.

Archaeal 16S rRNA clone sequences can be retrieved through the following GenBank accession numbers: *Methanobacterium petrolearium* (Mori & Harayama, 2011) KT936379, *Methanoculleus marisnigri* (Anderson et al., 2009) KT936380, *Methanoculleus receptaculi* (Cheng et al., 2008) KT936381, *Methanoculleus* sp. KT936385, *Methanomethylovorans hollandica* (Lomans et al., 1999) KT936389, *Methanosaeta concilii* (Barber et al., 2011) KT936386, *Methanosaeta harundinacea* (Ma, Liu, & Dong, 2006) KT936388, *Methanosarcina mazei* (Deppenmeier et al., 2002) KT936378, *Methanosarcina thermophila* (Zinder, Sowers, & Ferry, 1985) KT936384, *Methanosarcina vacuolata* (Maestrojuán & Boone, 1991) KT936383, *Methanosarcina* sp. KT936382.

## 3 Results

Four propionate‐degrading consortia (Ap1a, G12, N12, and Wp2a) were obtained from the fermenter sludge from four different agricultural biogas plants. The consortia were maintained via batch cultivation in biomass medium for years. The consortia were investigated in an 8‐week batch experiment for microbial succession during propionate degradation. The propionate concentration was monitored at the beginning of the experiment and also after 14, 39, and 56 days. At the latter three times, microbial samples were subjected to molecular 16S rRNA gene community analysis, revealing changing species compositions during propionate degradation. Furthermore, the total bacterial and archaeal cell titer of the samples were analyzed using quantitative PCR.

Table 1 presents the data concerning the propionate concentrations and cell titers. It shows that three (Ap1a, N12, and Wp2a) of the four consortia had degraded propionate significantly. Though formerly capable, consortium G12 had, for unknown reasons, failed to do so and was, therefore, considered as a negative consortium. Consortia Ap1a and N12 had degraded propionate completely and Wp2a had only 1 mmol L^−1^ remaining after 8 weeks. All samples (t_0_–t_3_) of the four consortia showed bacterial cell titer in the same order of magnitude (10^8^ cells/ml). However, the cell counts of negative consortium G12 lay below those of the positive consortia. Consortium G12 also showed the lowest archaeal cell titer after 14 and 39 days, being one order of magnitude below the titer of the positive consortia.

**Table 1:** Successive propionate degradation and cell titers of the consortia Ap1a, G12, N12, and Wp2a

	Samples of the consortia															
	Ap1a				G12				N12				Wp2a			
	t_0_	t_1_	t_2_	t_3_	t_0_	t_1_	t_2_	t_3_	t_0_	t_1_	t_2_	t_3_	t_0_	t_1_	t_2_	t_3_
Incubation [days]	0	14	39	56	0	14	39	56	0	14	39	56	0	14	39	56
Propionate [mmol L^−1^]	25	21	8	0	26	28	27	24	27	25	19	0	25	16	17	1
Archaea [cells ml^−1^]	—	1.2E^8^	1.1E^8^	1.5E^8^	—	1.2E^6^	4.6E^7^	5.3E^7^	—	2.0E^7^	1.6E^8^	3.0E^8^	—	2.7E^7^	1.1E^8^	3.8E^7^
Bacteria [cells ml^−1^]	—	5.5E^8^	3.9E^8^	5.0E^8^	—	1.5E^8^	1.7E^8^	1.8E^8^	—	2.5E^8^	5.1E^8^	6.9E^8^	—	1.5E^8^	8.7E^8^	5.5E^8^

Bacterial community analysis was conducted for three samples of each consortium, which were taken after 14, 39, and 56 days of incubation (samples t_1_–t_3_). Up to 60 bacterial 16S rRNA gene clones per sample were obtained and grouped via ARDRA. One 16S rRNA gene clone out of every ARDRA group was sequenced. These 16S rRNA gene sequences were clustered with 97% sequence identity resulting in thirteen sequence clusters, which dominated the bacterial diversity of the consortia. They were detectable either in all three samples of a consortium (samples t_1_–t_3_), or in one sample with at least 8% proportion of bacterial diversity. These dominating sequence clusters and their respective species relations are presented in Table 2. Six of these 16S rRNA sequence clusters showed more than 97% 16S rRNA gene sequence identity to closely related species and could, therefore, be identified on the species level, that is, *Defluviitoga tunisiensis*,* Mesotoga infera* (both Thermotogae), *Aminobacterium colombiense* (Synergistetes), *Tepidanaerobacter acetatoxydans* (Firmicutes), *Desulfovibrio aminophilus,* and *Syntrophobacter sulfatireducens* (both Proteobacteria). Due to lower sequence identities, four species were assigned on the genus level, that is, “*Candidatus* Cloacamonas sp.” (unclassified), *Treponema* sp. (Spirochaetes), *Ornatilinea* sp. (Chloroflexi), and *Sedimentibacter* sp. (Firmicutes). In three cases (*Cryptanaerobacter* sp./*Pelotomaculum* sp., *Caloramator* sp./*Moorella* sp., and *Syntrophaceticus* sp./*Thermacetogenium* sp., all Firmicutes), 16S rRNA gene sequence clusters consisted of two different, yet very closely related genera.

**Table 2:** Successive number of bacterial 16S rRNA gene clones of the consortia Ap1a, G12, N12, and Wp2a after 14 (t_1_), 39 (t_2_), and 56 (t_3_) days of incubation in propionate‐containing biomass medium

	Number of clones											
	Ap1a			G12			N12			Wp2a		
Phylogenetic relationship	t_1_	t_2_	t_3_	t_1_	t_2_	t_3_	t_1_	t_2_	t_3_	t_1_	t_2_	t_3_
Propionate‐oxidizing bacteria												
*Syntrophobacter sulfatireducens*		15	16									
Putative propionate‐oxidizing bacteria												
*Cryptanaerobacter* sp./*Pelotomaculum* sp.								14	26	16	33	27
*Candidatus* “Cloacamonas” sp.	2	3	2								1	9
Acetate‐oxidizing bacteria												
*Tepidanaerobacter acetatoxydans*	15	7	2	32	19	5	12	4	2	18	4	3
H_2_‐oxidizing bacteria												
*Desulfovibrio aminophilus*				4	8	24	7	3	1			
Putative H_2_‐oxidizing bacteria												
*Caloramator* sp./*Moorella* sp.							1	1	2			
*Syntrophaceticus* sp./*Thermacetogenium* sp.^a^								13	2			
Propionate‐forming bacteria												
*Aminobacterium colombiense*	24	6	20				1	2	1	4	7	2
Putative propionate‐forming bacteria												
*Sedimentibacter* sp.							6		1			
Sugar‐metabolizing bacteria												
*Defluviitoga tunisiensis*							2	1	1	2	2	1
*Mesotoga infera*	1	1	3	1	2	2				1	2	1
Putative sugar‐metabolizing bacteria												
*Ornatilinea* sp.										1	1	1
*Treponema* sp.	11	14	2	14	15	6	13	6	3			

a Also putative acetate‐oxidizing.

With respect to the physiological characteristics of the phylogenetic relationships, the species identified were allocated into five functionally different groups: propionate‐oxidizing, acetate‐oxidizing, hydrogen‐oxidizing, propionate‐forming, and sugar‐metabolizing bacteria, which are presented below.

### 3.1 Propionate‐oxidizing bacteria

Consortium Ap1a included *Syntrophobacter sulfatireducens*, a sulfate‐reducing *δ*‐proteobacterium, known for its syntrophic propionate‐oxidizing activity (Chen et al., 2005). Its proportion of bacterial diversity rose in the course of progressing propionate degradation (Table 2). “*Candidatus* Cloacamonas sp.” was also affiliated with propionate degradation. Its nearest species relation was “*Candidatus* Cloacamonas acidaminovorans” (92%–93% sequence identity), a so far uncultivated but genomically analyzed species, whose genome featured all the genes involved in propionate oxidation (Pelletier et al., 2008). It showed a considerable propagation in consortium Wp2a (Table 2) and might, therefore, have been involved in the propionate degradation of this consortium. A potentially propionate‐oxidizing key species of consortia N12 and Wp2a was *Cryptanaerobacter* sp./*Pelotomaculum* sp., whose sequences were related to *Cryptanaerobacter phenolicus*,* Pelotomaculum isophthalicum,* and *Pelotomaculum schinkii*, which are closely related species (Ezaki, 2009). The latter was described as a syntrophic propionate‐oxidizing species (De Bok et al., 2005). As the sequences did not exceed 97% sequence identity to any of the three species, it might have been a so far unknown species.

### 3.2 Acetate‐oxidizing bacteria

Acetate is a substrate for acetoclastic methanogenesis, and syntrophic acetate‐oxidizing bacteria (SAOB) can also be involved in acetate consumption. Four SAOB have been isolated and characterized to date: *Tepidanaerobacter acetatoxydans*,* Syntrophaceticus schinkii*,* Thermacetogenium phaeum,* and *Thermotoga lettingae* (Balk, Weijma, & Stams, 2002; Hattori, Kamagata, Hanada, & Shoun, 2000; Westerholm, Roos, & Schnurer, 2010; Westerholm et al., 2011). *Tepidanaerobacter acetatoxydans* was profoundly abundant throughout this analysis and could be detected in all samples (t_1_–t_3_) of the four consortia (Table 2). Its proportion of the species composition declined constantly during cultivation in all four consortia. Its potential function in propionate degradation could be its capability to degrade acetate in syntrophy with hydrogenotrophic archaea, forming H_2_ and CO_2_ under very low hydrogen partial pressure. This species was also prevalent in negative consortium G12, possibly feeding on complex substrates of the added biomass filtrate or its degradation products (e.g., also acetate). In addition, consortium N12 exhibited a putative SAOB, whose 16S rRNA gene sequence was closely related to *Syntrophaceticus schinkii* and *Thermacetogenium phaeum*, however, it has below 97% 16S rRNA gene sequence identity.

### 3.3 Hydrogen‐oxidizing bacteria

H_2_ consumption is essential for propionate degradation, due to its endergonic nature under elevated hydrogen partial pressure. Hydrogenotrophic methanogens, sulfate‐reducing bacteria and autotrophic homoacetogenic bacteria compete for H_2_ in methanogenic environments (Weijma et al., 2002). Sulfate‐reducing and H_2_/CO_2_‐using *Desulfovobrio aminophilus* (Baena, Fardeau, Labat, Ollivier, Garcia et al.,1998a) was found in positive consortium N12 (proportion decreasing) and negative consortium G12 (proportion increasing). Due to closely related autotrophic homoacetogenic *Moorella thermoacetica* and *Thermacetogenium phaeum* (Hattori, Galushko, Kamagata, & Schink, 2005; Pierce et al., 2008), *Caloramator* sp./*Moorella* sp. and *Syntrophaceticus* sp./*Thermacetogenium* sp. (consortium N12, Table 2) were considered as potential hydrogen consumers. Autotrophic homoacetogenesis (AHA) from H_2_/CO_2_ is the reverse reaction to the syntroph acetate oxidation (SAO) mentioned above. *Thermacetogenium phaeum* is even able to perform the reaction in both directions (Hattori et al., 2005) and was, therefore, mentioned above already. The potential role of AHA in propionate degradation may be the disposal of H_2_ under rising H_2_ partial pressure (e.g., if H_2_ consumption drops behind H_2_ formation).

### 3.4 Propionate‐forming bacteria

Since the positive consortia (Ap1a, N12, and Wp2a) degraded propionate efficiently, it was not surprising to find species which are able to form propionate. *Aminobacterium* *colombiense* is known for its syntrophic amino acid metabolism in coculture with methane‐forming hydrogenotrophic methanogens. Syntrophic glutamate and *α*‐ketoglutarate oxidation resulting in propionate formation were observed (Baena, Fardeau, Labat, Ollivier, Thomas et al., 1998b). Interestingly, within our analysis, *A. colombiense* was detected as a main cluster only in successfully propionate‐degrading consortia (Table 2). As transcriptomic analysis revealed potential amino acid transfer in syntrophic propionate‐oxidizing cocultures (Kato et al., 2009; Sieber et al., 2012), *A. colombiense* might be affiliated in this respect. The nearest species relations of *Sedimentibacter* sp. are *S. hydroxybenzoicus* and *S. saalensis*. These two species form propionate from acetate and pyruvate, respectively. They are involved in amino acid degradation as much as *A. colombiense* (Breitenstein et al., 2002; Zhang, Mandelco, & Wiegel, 1994).

### 3.5 Sugar‐metabolizing bacteria

*Mesotoga infera*,* Defluviitoga tunisiensis*,* Treponema* sp., and *Ornatilinea* sp. (Table 2) could not be linked to propionate degradation or formation directly. However, the former two species and the nearest species relations of the latter two share the trait of diverse sugar metabolism (Abt et al., 2013; Ben Hania et al., 2012, 2013; Podosokorskaya, Bonch‐Osmolovskaya, Novikov, Kolganova, & Kublanov, 2013; Pohlschroeder, Leschine, & Canale‐Parola, 1994). Interestingly, an ecogenomic analysis of a methanogenic bioreactor linked the genus *Mesotoga* to the syntrophic acetate oxidation mentioned above and found a Chloroflexi relative (such as *Ornatilinea*), apparently capable of H_2_‐oxidizing homoacetogenesis mentioned already (Nobu et al., 2015). Additionally, the nearest species relationships of the *Treponema* sp. sequences were close to *T. primitia*, an autotrophic homoacetogenic spirochete from termite hindguts (Graber & Breznak, 2004; Graber, Leadbetter, & Breznak, 2004). Though rather speculative, *Mesotoga infera*,* Treponema* sp., and *Ornatilinea* sp. might have been involved in the conversion of acetate to H_2_/CO_2_ and vice versa.

### 3.6 Methanogenic archaea

Archaeal species compositions of the four consortia were determined for samples t_2_ after 39 d of incubation. Up to 12 archaeal 16S rRNA gene clones were analyzed as the species diversity was expected to be substantially lower compared to bacterial diversity (Table 3). Archaeal compositions differed according to potentially propionate‐degrading key species. *Syntrophobacter sulfatireducens* was detected in the presence of hydrogenotrophic *Methanoculleus* as well as acetoclastic *Methanosaeta* in consortium Ap1a. In contrast, *Cryptanaerobacter* sp./*Pelotomaculum* sp. grew with *Methanosarcina mazei* and *Methanosarcina vacuolata* in consortia N12 and Wp2a, respectively. These *Methanosarcina* species are able to utilize all propionate oxidation end products, H_2_, CO_2_, and acetate (Maestrojuán & Boone, 1991). Negative consortium G12 was dominated by the hydrogenotrophic species *Methanoculleus receptaculi* and *Methanoculleus marisnigri*.

**Table 3:** Composition of archaeal 16S rRNA gene clones of the consortia Ap1a, G12, N12, and Wp2a after 39 days of incubation

Phylogenetic relationship		Number of clones		
	Ap1a	G12	N12	Wp2a
Acetoclastic and hydrogenotrophic methanogenesis				
*Methanosarcina mazei*			10	
*Methanosarcina thermophila*				1
*Methanosarcina vacuolata*				9
*Methanosarcina* sp.				1
Acetoclastic methanogenesis only				
*Methanosaeta concilii*	1			
*Methanosaeta harundinaceae*	1			
Hydrogenotrophic methanogenesis only				
*Methanobacterium petrolearium*			1	
*Methanoculleus marisnigri*		2		
*Methanoculleus receptaculi*	4	5		
*Methanoculleus* sp.	1			
Neither acetoclastic nor hydrogenotrophic methanogenesis				
*Methanomethylovorans hollandica*	1			

## 4 Discussion

We investigated the successive microbial composition of four propionate‐degrading consortia (Ap1a, N12, G12, and Wp2a) during propionate degradation. The consortia were cultivated in an 8‐week batch experiment. Microbial samples were taken after 14, 39, and 56 days of incubation and analyzed via parallel molecular 16S rRNA gene community analysis.

Investigations concerning anaerobic propionate degradation community structures have been conducted for rice field soil and municipal and molasses wastewater. The propionate‐oxidizing bacteria identified belonged to the genera *Pelotomaculum*,* Syntrophobacter,* and *Smithella*. Variable methanogenic compositions were detected (Ariesyady et al., 2007; Ban et al., 2013, 2015; Gan et al., 2012; Lueders et al., 2004). The genera *Methanobacterium* and *Methanosarcina* dominated the archaeal community during propionate degradation by flooded rice field soil samples (Lueders et al., 2004), a result which we also observed within consortium N12. Moreover, our results suggest that *Methanosarcina* species (*M*. *mazei*,* M*. *vacuolata*) grow preferably along with propionate‐oxidizing species of the genus *Pelotomaculum*. In contrast, acetoclastic *Methanosaeta* and hydrogenotrophic *Methanospirillum* were the dominant methanogenic genera in an upflow anaerobic sludge blanket reactor running on molasses wastewater (Ban et al., 2013). A similar composition propagated within our propionate‐degrading consortium Ap1a, whose propionate‐degrading key species was *Syntrophobacter sulfatireducens*. Here, *Methanosaeta concilii* and *Methanosaeta harundinacea* were found with *Methanoculleus receptaculi*, whose electron donor usage is identical to that from *Methanospirillum* spp. (Kim & Gadd, 2008). Furthermore, our studies reveal, that genetically putative propionate‐oxidizing Cloacimonete “*Candidatus* Cloacamonas sp.” (Pelletier et al., 2008) actually propagates in propionate‐degrading communities.

In addition to the identification of the propionate‐oxidizing and methanogenic key species, our goal was to identify further bacterial species which might be part of the propionate degradation community, but have been hitherto neglected. With respect to our findings, acetate‐ and H_2_‐consuming bacteria came under consideration. The ubiquitous occurrence of the syntrophic acetate‐oxidizing species *Tepidanaerobacter acetatoxydans* and the detection of putative autotrophic homoacetogenic *Moorella* and *Thermacetogenium*‐related species, as well as further genera, which can be linked to SAO (*Syntrophaceticus*,* Mesotoga*) and AHA (*Treponema*), indicate an involvement of SAO and AHA in propionate degradation. Although repeatedly detected in methanogenic ecosystems, information about the ecological roles of SAO and AHA are currently limited (Saady, 2013; Westerholm, Leven, & Schnurer, 2012).

$${\text{Acetate}}^{-}+4H_{2}O\to 2{\text{HCO}}_{3}^{-}+4H_{2}+H^{+}$$

$${\text{2HCO}}_{3}^{-}+{\text{4H}}_{2}+H^{+}\to {\text{Acetate}}^{-}+{\text{4H}}_{2}O$$

**Figure 1:**
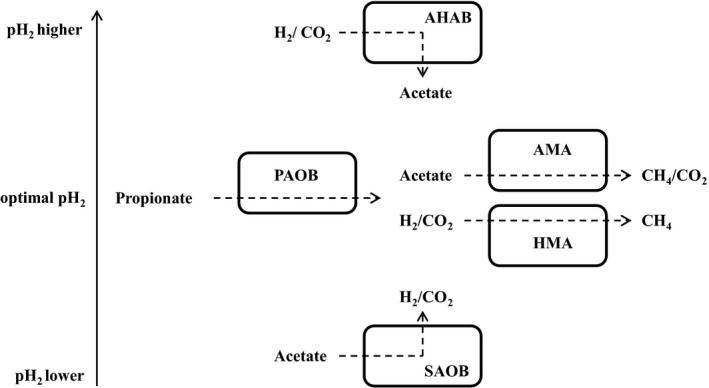
Hypothetical stabilization of anaerobic propionate degradation by hydrogen partial pressure adjusting bacteria. AHAB: autotrophic homoacetogenic bacteria, AMA: acetoclastic methanogenic archaea, HMA: hydrogenotrophic methanogenic archaea, PAOB: propionate‐oxidizing bacteria, SAOB: syntrophic acetate‐oxidizing bacteria

Since this reaction can act as a sink as well as a source of hydrogen, it offers the potential to adjust and stabilize the hydrogen partial pressure in anaerobic biomass digestion systems, such as syntrophic propionate degradation in biogas plants. Regarding the Gibbs free energy of propionate oxidation, SAO, AHA, acetoclastic, and hydrogenotrophic methanogenesis (Table 4), it is noticeable that SAO and AHA will not occur if acetoclastic and hydrogenotrophic methanogenesis are equally efficient (at 5 × 10^−5^ atm pH_2_). However, if pH_2_ increases or decreases significantly, propionate oxidation or hydrogenotrophic methanogenesis, respectively, lose free energy (Table 4), most probably resulting in propionate degradation instability due to product formation/disposal imbalance. Therefore, SAO and AHA may counterbalance severe hydrogen input, excess hydrogen formation or hydrogen deficiency, leading to increased process balance and stability (Fig. 1). Neither AHA nor SAO reduce the methane yield, because either product serves as a methanogenic precursor. Furthermore, AHA and SAO performing species (e.g., *Moorella thermoacetica*,* Tepidanaerobacter acetatoxydans*) can be competent sugar metabolizers (Pierce et al., 2008; Westerholm et al., 2011), which do not depend on the low energy yield of AHA or SOA at low pH_2_; however, they depend on a stable biotope with efficient propionate degradation and biogas formation. In conclusion, stable and efficient propionate degradation might rely not only on propionate oxidation, acetoclastic, and hydrogenotrophic methanogenesis, but also on pH_2_‐adjusting SAO and AHA.

**Table 4:** Gibbs free energy calculations of anaerobic metabolic reactions according to Zinder, 1984, conducted for variable hydrogen partial pressures (37°C, 1 mmol L^−1^ acetate and propionate, 20 mmol L^−1^ HCO_3_^−^, 0.6 atm CH_4_)

pH_2_ [atm]	Propionate oxidation [kJ/reaction]	Hydrogenotrophic methanogenesis [kJ/reaction]	Acetoclastic methanogenesis [kJ/reaction]	SAO^a^ [kJ/reaction]	AHA^b^ [kJ/reaction]
10^−1^	48	−103	−25	78	−78
10^−2^	30	−79	−25	55	−55
10^−3^	13	−56	−25	31	−31
10^−4^	−5	−32	−25	7	−7
5 × 10^−5^	−11	−25	−25	0	0
10^−5^	−23	−8	−25	−17	17
10^−6^	−41	16	−25	−40	40
10^−7^	−59	39	−25	−64	64

a Syntrophic acetate oxidation.

b Autotrophic homoacetogenesis.

## Funding Information

This study was funded through research grants from the German Federal Ministry of Education and Research (03EK3526C).

## Conflict of Interest

None declared.
